# A Robust Diffusion Minimum Kernel Risk-Sensitive Loss Algorithm over Multitask Sensor Networks

**DOI:** 10.3390/s19102339

**Published:** 2019-05-21

**Authors:** Xinyu Li, Qing Shi, Shuangyi Xiao, Shukai Duan, Feng Chen

**Affiliations:** 1 College of Artificial Intelligence, Southwest University, Chongqing 400715, China; lxyv5@email.swu.edu.cn; 2 Key Laboratory of Nonlinear Circuits and Intelligent Information Processing, and College of Electronic and Information Engineering, Southwest University, and Chongqing Collaborative Innovation Center for Brain Science, Chongqing 400715, China; shiqing@email.swu.edu.cn (Q.S.); xsy2016@email.swu.edu.cn (S.X.); duansk@swu.edu.cn (S.D.)

**Keywords:** distributed estimation, diffusion minimum kernel risk-sensitive loss, multitask, impulsive noise, sensor networks

## Abstract

Distributed estimation over sensor networks has attracted much attention due to its various applications. The mean-square error (MSE) criterion is one of the most popular cost functions used in distributed estimation, which achieves its optimality only under Gaussian noise. However, impulsive noise also widely exists in real-world sensor networks. Thus, the distributed estimation algorithm based on the minimum kernel risk-sensitive loss (MKRSL) criterion is proposed in this paper to deal with non-Gaussian noise, particularly for impulsive noise. Furthermore, multiple tasks estimation problems in sensor networks are considered. Differing from a conventional single-task, the unknown parameters (tasks) can be different for different nodes in the multitask problem. Another important issue we focus on is the impact of the task similarity among nodes on multitask estimation performance. Besides, the performance of mean and mean square are analyzed theoretically. Simulation results verify a superior performance of the proposed algorithm compared with other related algorithms.

## 1. Introduction

Distributed data processing over sensor networks has emerged as an attractive and challenging research area for various applications such as industrial automation, cognitive radios and inference tasks [1,2,3,4]. Distributed estimation plays a significant role in distributed data processing, which estimates some parameters of interest from noise measurements by exchanging information with neighboring nodes. Most algorithms proposed for distributed estimation can be classified into a consensus strategy [5,6,7,8], incremental strategy [9,10,11] and diffusion strategy [12,13,14]. In our work, we center on a diffusion strategy, which is robust, fully distributed and flexible among these strategies [15,16,17,18,19].

Diffusion strategies are particularly attractive schemes in distributed estimation, such as diffusion Recursive Least Squares (RLS) [20,21], diffusion Least Mean Square (LMS) [13,14]. With the mean-square error (MSE) criterion, these algorithms can accomplish a satisfying performance in a Gaussian noise environment. However, their performance may deteriorate dramatically in the presence of impulsive noise [22,23]. Some algorithms have been proposed to solve the issue, including Diffusion least-mean power (D-LMP) and the Diffusion sign-error Least Mean Square (DSE-LMS) adaptive filtering algorithm [24,25]. To efficiently address the non-Gaussian noise, the correntropy [26,27] was proposed, which is a higher order statistic and widely used in adaptive filters. Moreover, the generalized maximum correntropy criterion (GMCC) algorithm and the minimum kernel risk-sensitive loss (MKRSL) were proposed [28,29], which provide more general frameworks and better performance. In this work, we consider the diffusion minimum kernel risk-sensitive loss (D-MKRSL) algorithm for distributed estimation over multitask networks.

In previous works, diffusion strategies mainly focus on the single-task estimation problem where an identical parameter vector is estimated by all the nodes [30]. On the contrary, many essential applications are multitask-oriented, such as regression, web page categorization and target location tracking. In the above situations, multiple optimum vectors are different but related, which are inferred synchronously over the networks by all nodes in a collaborative manner. This type of problem is known as a multitask problem. Generally, distributed estimation problems over multitask networks can be roughly classified into two fields. In the first case, there is no knowledge about the correlation of tasks. Meanwhile, which nodes share the same tasks is unknown and nodes cooperate according to network topology [31,32,33]. In the second situation, it is assumed that nodes know which cluster they belong to and the parameter vector in each cluster is the same. Exploiting the information about the similarity of tasks, diffusion strategies for distributed estimation over multitask are obtained [34,35,36,37]. In our work, we focus on the second case.

Inspired by the adapt-then-combine (ATC) DLMS algorithm, we propose the diffusion MKRSL algorithm over multitask networks. The algorithm can achieve desirable performance in both Gaussian and impulsive noise environments. Additionally, the impact of task relatedness on estimation performance is also studied. Moreover, the mean and mean square stability are analyzed theoretically. Effectiveness and advantages of the proposed algorithm are verified by simulation results.

The remaining parts of the article are organized as follows: In Section 2, we briefly introduce the data model of distributed estimation and propose the multitask Diffusion MKRSL algorithm. In Section 3, the mean and mean square performance of the multitask D-MKRSL algorithm are analyzed. Simulation results are demonstrated in Section 4. Finally, we draw conclusions in Section 5.

*Notation:* We use ${\left(.\right)}^{T}$, $E\left[.\right]$ and ⨂ to denote transposition, expectation and Kronecker product operators, respectively. $I_{m}$ denotes an m×m identity matrix. 1 is an N×1 all-unity vector. $\left|.\right|$ is the absolute value of a scalar.

## 2. Multitask Diffusion Estimation

### 2.1. Data Model

Let us consider a connected network with *K* nodes. Every node $k\in \left\{1,2,\ldots ,K\right\}$ has access to scalar random variables $d_{k,i}$ and a zero-mean M×1 regression vector $u_{k,i}$ at every time instant i≥0. The data of node *k* is related via the linear regression model:

(1) $$d_{k,i}=u_{k,i}^{T}w_{k}^{0}+n_{k,i}$$

where $n_{k,i}$ is the random measurement noise with zero-mean and variance $\sigma_{n,k}^{2}$, which is independent of regression vector $u_{k,i}$. The goal of distributed estimation is to estimate an M×1 deterministic but unknown vector $w_{k}^{0}$ by exchanging and combining the data only from neighboring nodes, which is regarded as single-task problem with $w_{k}^{0}=w^{0}$ for k=1,2,…,K, and multitask problem with $w_{k}^{0}\neq w_{l}^{0}$ for k≠l. It is assumed that there is no limit to how much information can be transmitted among neighbors.

### 2.2. Diffusion MKRSL Algorithm

In many previous works, the diffusion distributed estimation algorithms are based on the MSE criterion, which achieves desirable performance if the measurement noise is Gaussian, while their performance may deteriorate dramatically in an impulsive noise environment. To solve the parameter estimation problem over multitask sensors networks, it becomes a significant focus of our interest to design a novel algorithm that is robust to both Gaussian noises and impulsive noises.

The information theoretic learning (ITL) plays a significant role and provides a general framework in distributed parameter estimation for non-Gaussian cases. The correntropy is a local statistical similarity measure in ITL, which is defined by Reference [26]

(2) $$V\left(X,Y\right)=E\left[k_{\sigma }\left(X-Y\right)\right]=\int k_{\sigma }\left(x-y\right)dF_{XY}\left(x,y\right)$$

where X,Y are two random variables, $k_{\sigma }\left(.\right)$ is a shift-invariant Mercer kernel and σ>0 denotes the kernel bandwidth. $F_{XY}\left(x,y\right)$ is the joint distribution function of $\left(X,Y\right)$. In our work, we focus on the Gaussian kernel, which takes the following form:

(3) $$k_{\sigma }\left(x-y\right)=\exp\left(-\frac{{\left(x-y\right)}^{2}}{2\sigma^{2}}\right)$$

The minimum kernel risk-sensitive loss (MKRSL) algorithm is derived by applying the KRSL to develop a new adaptive filtering algorithm, which shows better convex properties than correntropic loss on the error performance surface [29,38]. The KRSL between two random variables *X* and *Y* is defined by

(4) $$\begin{array}{ll} L_{\lambda }\left(X,Y\right) & =\frac{1}{\lambda }E\left[\exp\left(\lambda \left(1-k_{\sigma }\left(X-Y\right)\right)\right)\right]\\ & =\frac{1}{\lambda }\int \exp\left(\lambda \left(1-k_{\sigma }\left(X-Y\right)\right)\right)dF_{XY}\left(x,y\right) \end{array}$$

where λ>0 is the risk-sensitive parameter. Nevertheless, the exact joint distribution of $\left(X,Y\right)$ is usually unavailable in application scenarios. On the contrary, only a limited number of sample values ${\left\{x\left(i\right),y\left(i\right)\right\}}_{i=1}^{L}$ are known. Therefore, the sample mean estimator of KRSL—called empirical KRSL—is calculated by an average over samples:

(5) $${\hat{L}}_{\lambda }\left(X,Y\right)=\frac{1}{L\lambda }\sum_{i=1}^{L}\exp(\lambda \left(1-k_{\sigma }\left(x\left(i\right)-y\left(i\right)\right)\right))$$

Then, the KRSL cost function is derived as

(6) $$J_{KRSL}=\frac{1}{L\lambda }\sum_{i=1}^{L}\exp(\lambda \left(1-k_{\sigma }\left(e\left(i\right)\right)\right))$$

with $e\left(i\right)=d\left(i\right)-u_{i}^{T}w$. The time average of the KRSL cost function in the above equation can be replaced by the expectation

(7) $$J_{KRSL}^{\prime }=\frac{1}{\lambda }E\left[\exp\left(\lambda \left(1-k_{\sigma }\left(e\left(i\right)\right)\right)\right)\right]$$

Based on the KRSL cost function mention in the above Equation(7), the instantaneous cost function of the KRSL algorithm is approximated as

(8) $${\tilde{J}}_{KRSL}=\frac{1}{\lambda }\exp\left(\lambda \left(1-k_{\sigma }\left(e\left(i\right)\right)\right)\right)$$

For the distributed diffusion estimation problem, our goal is to seek the best $w_{k}^{0}$ by minimizing the diffusion KRSL cost function at each node *k* by cooperating with all neighboring nodes. For each node *k*, $N_{k}$ is the one-hop neighbor set and $\left\{c_{l,k}\right\}$ are non-negative real cooperative according to Metropolis rule weights satisfying

(9) $$c_{l,k}=\left\{\begin{array}{l} \frac{1}{\max\left(n_{k,}n_{l}\right)},\;\mathrm{if}\;\;l\in N_{k}\setminus k,\\ 1-\sum_{l\in N_{k}\setminus k}c_{l,k},\;\mathrm{if}\;\;l=k,\\ 0.\;\;\;\;\;\;\;\;\;\;\;\mathrm{if}\;l\notin N_{k}, \end{array}\right.$$

where $n_{k}$ is the degree of node *k*. The real, non-negative combining coefficients $c_{l,k}$ satisfy the following conditions: $\sum_{l\in N_{k}\cup k}c_{l,k}=1$ and $c_{l,k}=0\;\;if\;\;l\notin N_{k},\;CI=I,\;I^{T}C=I^{T}$, where C is an N×N matrix. The KRSL local cost function at each node *k* can be formulated as

(10) $$\begin{array}{ll} J_{k}^{loc}\left(w\right) & =\sum_{l\in N_{k}}c_{l,k}{\tilde{J}}_{KRSL}\left(e_{l,i}\right)\\ & =\frac{1}{\lambda }\sum_{l\in N_{k}}c_{l,k}\exp\left(\lambda \left(1-k_{\sigma }\left(e_{l,i}\right)\right)\right)\\ & =\frac{1}{\lambda }\sum_{l\in N_{k}}c_{l,k}\exp\left(\lambda \left(1-k_{\sigma }\left(d_{l,i}-u_{l,i}^{T}w\right)\right)\right) \end{array}$$

Based on the KRSL local cost function, the derivative of (10) with respect to *w* can be derived as

(11) $$\begin{array}{ll} \nabla J_{k}^{loc}\left(w\right) & =\frac{1}{\lambda }\sum_{l\in N_{k}}c_{l,k}\frac{\partial }{\partial w}\exp\left(\lambda \left(1-k_{\sigma }\left(e_{l,i}\right)\right)\right)\\ & =-\frac{1}{\sigma^{2}}\sum_{l\in N_{k}}c_{l,k}\exp\left(\lambda \left(1-k_{\sigma }\left(e_{l,i}\right)\right)\right)k_{\sigma }\left(e_{l,i}\right)e_{l,i}u_{l,i}^{T} \end{array}$$

At node *k*, the weight vector update equation based on a stochastic gradient for $w_{k}^{0}$ is obtained by

(12) $$\begin{array}{ll} w_{k}\left(i\right) & =w_{k}\left(i-1\right)-\mu \nabla J_{k}^{loc}\left(w\right)\\ & =w_{k}\left(i-1\right)+\frac{\mu }{\sigma^{2}}\sum_{l\in N_{k}}c_{l,k}\exp\left(\lambda \left(1-k_{\sigma }\left(e_{l,i}\right)\right)\right)k_{\sigma }\left(e_{l,i}\right)e_{l,i}u_{l,i}^{T}\\ & =w_{k}\left(i-1\right)+\eta \sum_{l\in N_{k}}c_{l,k}\exp\left(\lambda \left(1-k_{\sigma }\left(e_{l,i}\right)\right)\right)k_{\sigma }\left(e_{l,i}\right)e_{l,i}u_{l,i}^{T} \end{array}$$

where $\eta =\frac{\mu }{\sigma^{2}}$ is step-size and $w_{k}\left(i\right)$ is estimator for $w_{k}^{0}$ at time index *i*. The above algorithm is a new expression of the MKRSL algorithm. Inspired by the general framework for a diffusion-based distributed estimation algorithm [13], an adapt-then-combine (ATC) strategy for a diffusion MKRSL algorithm is proposed. The ATC scheme first updates the value of the estimator for each node with the adaptive algorithm. Then, the intermediate estimates are fused from its neighbors for each node *k*. The intermediate estimate at each node *k* is defined as:

(13) $$\varphi_{k}\left(i-1\right)=\sum_{l\in N_{k}}\beta_{l,k}w_{l}\left(i-1\right)$$

The nodes update their intermediate estimates by

(14) $$\varphi_{k}\left(i\right)=\varphi_{k}\left(i-1\right)+\eta \sum_{l\in N_{k}}c_{l,k}\exp\left(\lambda \left(1-k_{\sigma }\left(e_{l,i}\right)\right)\right)k_{\sigma }\left(e_{l,i}\right)e_{l,i}u_{l,i}^{T}$$

$\varphi_{k}\left(i-1\right)$ is an intermediate estimate at time index i−1 for node *k*. The non-negative real value $\beta_{l,k}$ is a weight coefficient, which corresponds to the matrices B, especially B=I in the ATC scheme [12]. Therefore, we can obtain:

(15) $$\varphi_{k}\left(i\right)=w_{k}\left(i-1\right)+\eta \sum_{l\in N_{k}}c_{l,k}\exp\left(\lambda \left(1-k_{\sigma }\left(e_{l,i}\right)\right)\right)k_{\sigma }\left(e_{l,i}\right)e_{l,i}u_{l,i}^{T}$$

In the above Equation (15), the task relatedness among nodes is ignored, which is called non-cooperative diffusion MKRSL in this article.

However, multitask estimation is an attracting filed in practical applications. Nodes are grouped into some clusters and each cluster has an identical task in clustered multi-task networks. Furthermore, utilizing the relatedness of tasks, the performance of distributed estimation can be improved. The Equation (15) is adjusted for multitask estimation:

(16) $$\varphi_{k}\left(i\right)=w_{k}\left(i-1\right)+\eta \sum_{l\in N_{k}\cap c\left(k\right)}c_{l,k}\exp\left(\lambda \left(1-k_{\sigma }\left(e_{l,i}\right)\right)\right)k_{\sigma }\left(e_{l,i}\right)e_{l,i}u_{l,i}^{T}+\tau \sum_{l\in N_{k}\setminus c\left(k\right)}\rho_{kl}\left(w_{l}\left(i\right)-w_{k}\left(i\right)\right)$$

$c\left(k\right)$ is the cluster of node *k*, with the cluster of node *k* non-negative strength parameter τ, weights $\rho_{kl}$ and $\eta \left(i\right)=\exp\left(\lambda \left(1-k_{\sigma }\left(e_{i}\right)\right)\right)k_{\sigma }\left(e_{i}\right)$. The notation $N_{k}\cap c\left(k\right)$ is the set of neighboring nodes *k* and in the same cluster as *k*. On the contrary, $N_{k}\setminus c\left(k\right)$ denotes the set of neighboring nodes of *k* that are not in the same cluster as *k*. The Equations (15) and (16) are defined as the increment step. The combination step can then be derived as

(17) $$w_{k}\left(i\right)=\sum_{l\in N_{k}}c_{l,k}\varphi_{l}\left(i\right)$$

**Figure 1:**
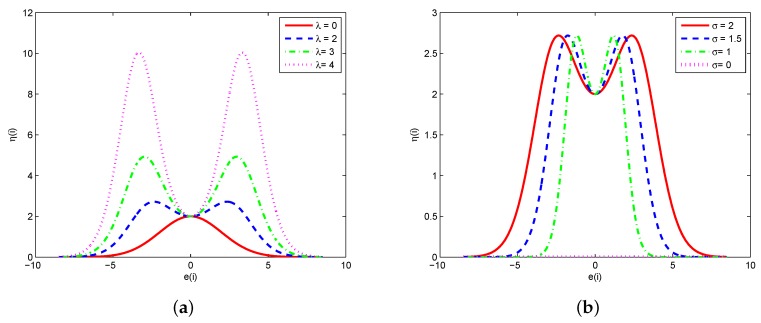
Curves of η(i) as a function of e(i) (**a**) different values of λ(σ=η=2.0) (**b**) different values of σ(λ=η=2.0).

The step-size η(i) is a function of e(i) and the curves with different values of λ (where σ=η=2.0) and σ (where λ=η=2.0) is depicted in Figure 1.

It is shown that the step-size η(i) will approach zero as $\left|e(i)\right|\to \infty$ for different values of λ. Therefore, the MKRSL algorithm maintains the robustness to outliers, such as impulsive noise.

For a better understanding, the Multitask Diffusion MKRSL algorithm is summarized in Algorithm 1:
**Algorithm 1:** Multitask Diffusion MKRSL Algorithm **Input:**
$d_{k,i}$, $u_{k,i}^{T}$, η, τ, and $\left\{c_{l,k}\right\}$ satisfying (10) **Initialization:** Start with $\left\{w_{l,-1}=0\right\}$ for all *l*. **for**
i=1:T    **for each node**
*k***:**    **Adaptation** $\;\varphi_{k}\left(i\right)=w_{k}\left(i-1\right)+\eta \sum_{l\in N_{k}\cap c\left(k\right)}c_{l,k}\exp\left(\lambda \left(1-k_{\sigma }\left(e_{l,i}\right)\right)\right)k_{\sigma }\left(e_{l,i}\right)e_{l,i}u_{l,i}^{T}$ $\;+\tau \sum_{l\in N_{k}\setminus c\left(k\right)}\rho_{kl}\left(w_{l}\left(i\right)-w_{k}\left(i\right)\right)$    **Communication**   **Transmit the intermediate**
$\varphi_{k}\left(i\right)$
**to all neighbors in**
$N_{k}$    **Combination**            $w_{k}\left(i\right)=\sum_{l\in N_{k}}c_{l,k}\varphi_{l}\left(i\right)$ **end for**


## 3. Performance Analysis

The multitask D-MKRSL algorithms are evaluated theoretically under model (1) in this section. In the following, some common assumptions are adopted for tractable analysis [39,40].

(1) The regression vector $u_{k,i}$ is independently and identically distributed (i.i.d.) and $E\left[u_{k,i}u_{k,i}^{T}\right]=R_{u,k}$.

(2) For each node *k* at time index *i*, the input noise $n_{k}\left(i\right)$ is independent of $u_{k,i}$ and is a mixture signal of zero mean Gaussian, we have $E[n_{k,i}]=0$.

(3) The step-size η is small enough, so the squared value can be negligible.

Then, the estimate-error vectors are defined as follows:

(18) $${\tilde{w}}_{k,i}=w_{k}^{0}-w_{k,i}$$

and

(19) $${\tilde{\varphi }}_{k,i}=w_{k}^{0}-\varphi_{k,i}$$

Furthermore, the global quantities are defined to covert the local variables to global ones:

(20) $$K=blockdiag\left\{\eta I_{M},\ldots ,\eta I_{M}\right\}$$

(21) $$X=blockdiag\left\{\tau I_{M},\ldots ,\tau I_{M}\right\}$$

(22) $${\tilde{w}}_{i}=col\left\{{\tilde{w}}_{1,i},\ldots ,{\tilde{w}}_{K,i}\right\}$$

(23) $$w_{i}=col\left\{w_{1,i},\ldots ,w_{K,i}\right\}$$

(24) $$w_{k}^{0}=col\left\{w_{1}^{0},\ldots ,w_{K}^{0}\right\}$$

### 3.1. Mean Performance

We consider the gradient error caused by replacing the cost function of KRSL with instantaneous values. The gradient error of the intermediate estimate at time *i* and each node *k* is defined as follows:

(25) $$s_{k}\left(w_{k,i-1}\right)={\hat{f}}_{k}\left(w_{k,i-1}\right)-f_{k}\left(w_{k,i-1}\right)$$

where ${\hat{f}}_{k}\left(w_{k,i-1}\right)=\frac{1}{\sigma^{2}}\exp\left(\lambda \left(1-k_{\sigma }\left(e_{k,i-1}\right)\right)\right)k_{\sigma }\left(e_{k,i-1}\right)e_{k,i-1}u_{k,i-1}^{T}$ and $f_{k}\left(w_{k,i-1}\right)=\frac{1}{\sigma^{2}}E\left[\exp\left(\lambda \left(1-k_{\sigma }\left(e_{k,i-1}\right)\right)\right)k_{\sigma }\left(e_{k,i-1}\right)e_{k,i-1}u_{k,i-1}^{T}\right]$

The update equation of the intermediate estimate can be rewritten as

(26) $$\varphi_{k,i}=w_{k,i-1}+\mu \left(s_{k}\left(w_{k,i-1}\right)+f_{k}\left(w_{k,i-1}\right)\right)$$

$f_{k}\left(w_{k,i-1}\right)$ is twice continuous differentiable in a neighborhood of a line segment between points $w_{k}^{0}$ and $w_{k-1}$. Thus, based on the Theorem 1.2.1 in Reference [41], we have

(27) $$f_{k}\left(w_{k,i-1}\right)=f_{k}\left(w_{k}^{0}\right)-\left(\int_{0}^{1}H_{k}\left(w_{k}^{0}-t{\tilde{w}}_{k,i-1}\right)dt\right){\tilde{w}}_{k,i-1}$$

where $H_{k}\left(w\right)$ is the Hessian matrix of $f_{k}\left(w_{k,i-1}\right)$. ${\tilde{w}}_{k,i-1}=w_{k}^{0}-w_{k,i-1}$ is the weight error vector for node *k*. The unknown vector $w_{k}^{0}$ is the real-value that we want to estimate, so $f_{k}\left(w_{k}^{0}\right)$ is equal to zero. The estimate of each node converges to the vicinity of the unknown vector $w_{k}^{0}$. Thus, ${\tilde{w}}_{k,i}$ is small enough such that it is negligible, yielding

(28) $$\begin{array}{ll} f_{k}\left(w_{k,i-1}\right) & \approx -\left(\int_{0}^{1}H_{k}\left(w_{k}^{0}\right)dt\right){\tilde{w}}_{k,i-1}\\ & =-H_{k}\left(w_{k}^{0}\right){\tilde{w}}_{k,i-1}\\ & =-\beta R_{u,k}{\tilde{w}}_{k,i-1} \end{array}$$

where $R_{u,k}=E\left[u_{k,i}u_{k,i}^{T}\right]$ and β is a constant.

So, the approximate value of the gradient error at the value of $w_{k}^{0}$ is

(29) $$\begin{array}{ll} s_{k}\left(w_{k,i-1}\right) & \approx s_{k}\left(w_{k}^{0}\right)\\ & ={\hat{f}}_{k}\left(w_{k}^{0}\right)-f_{k}\left(w_{k}^{0}\right)\\ & =\frac{1}{\sigma^{2}}\exp\left(\lambda \left(1-k_{\sigma }\left(e_{k,i-1}\right)\right)\right)k_{\sigma }\left(e_{k,i-1}\right)e_{k,i-1}u_{k,i-1}^{T} \end{array}$$

Substituting (28) and (29) into (26) and adjusting for multitask estimation, we can get the intermediate estimate

(30) $$\varphi_{k,i}=w_{k,i-1}+\mu \left(s_{k}\left(w_{k}^{0}\right)-H_{k}\left(w_{k}^{0}\right){\tilde{w}}_{k,i-1}\right)+\tau Q\left({\tilde{w}}_{k,i-1}+w_{k}^{0}\right)$$

where

(31) $$Q=I_{\mathrm{MN}}-P⊗I_{M}$$

P is the matrix with $\left(k,l\right)$-th entry $\rho_{kl}$. Substituting (30) into (17), we can get the update equation of $w_{k}\left(i\right)$ as follows

(32) $$w_{k}\left(i\right)=\sum_{l\in N_{k}}c_{l,k}\left[w_{k,i-1}+\mu \left(s_{k}\left(w_{k}^{0}\right)-H_{k}\left(w_{k}^{0}\right){\tilde{w}}_{k,i-1}\right)+\tau Q\left({\tilde{w}}_{k,i-1}+w_{k}^{0}\right)\right]$$

Define global quantity $H=diag\left\{H_{1}\left(w_{1}^{0}\right),\ldots ,H_{k}\left(w_{N}^{0}\right)\right\}$ and rewrite (32) as

(33) $$w_{i}=C\left(w_{i-1}+Ks_{i}-\mathrm{KH}{\tilde{w}}_{i-1}+\mathrm{XQ}{\tilde{w}}_{i-1}+\mathrm{XQ}w^{0}\right)$$

Noting that $Cw^{0}=w^{0}$, subtracting both sides of (33) from $w^{0}$, the global vector is obtained:

(34) $${\tilde{w}}_{i-1}=C\left(I_{\mathrm{MN}}-\mathrm{KH}+\mathrm{XQ}\right){\tilde{w}}_{i-1}+\mathrm{CK}s_{i}+\mathrm{CXQ}w^{0}$$

Calculating the expectation of (34) leads to

(35) $$E\left[{\tilde{w}}_{i-1}\right]=C\left(I_{\mathrm{MN}}-\mathrm{KH}+\mathrm{XQ}\right)E\left[{\tilde{w}}_{i-1}\right]+\mathrm{CK}E\left[s_{i}\right]+\mathrm{CXQ}w^{0}$$

where $E\left[s_{i}\right]=col\left\{E[s_{1}\left(w_{1}^{0}\right),\ldots ,s_{N}\left(w_{N}^{0}\right)]\right\}=0$. Based on Lemma 1 of [13], the matrix $I_{\mathrm{MN}}-\mathrm{KH}+X$ should be stable to guarantee mean stability. There it holds that

(36) $$\left|\lambda_{\max}\left(I_{\mathrm{MN}}-\mathrm{KH}+\mathrm{XQ}\right)\right|<1$$

$\lambda_{\max}$ is the largest eigenvalue of matrix. Thus, a sufficient condition for maintaining the stability of the algorithm is:

(37) $$0<\eta <\frac{2}{\beta \lambda_{\max}\left(R_{u,k}\right)+2\tau }$$

### 3.2. Mean-Square Performance

In this section, we mainly focus on the mean-square performance of the proposed algorithm. Computing the weight norm of (34) and calculating the expectations, we can obtain

(38) $$E\left[{∥{\tilde{w}}_{i}∥}_{\Sigma }^{2}\right]=E\left[{∥{\tilde{w}}_{i-1}∥}_{\Gamma }^{2}\right]+E\left[s_{i}^{T}KC^{T}\Sigma \mathrm{CK}s_{i}\right]+2{\left(\mathrm{XQ}w^{0}\right)}^{T}\Sigma C\left(I_{\mathrm{MN}}-\mathrm{KH}+\mathrm{XQ}\right)E\left[{\tilde{w}}_{i-1}\right]+\mathrm{CXQ}w^{0}$$

where

(39) $$\Gamma =\left(I_{\mathrm{MN}}-\mathrm{KH}+\mathrm{XQ}\right)C^{T}\Sigma C\left(I_{\mathrm{MN}}-\mathrm{KH}+\mathrm{XQ}\right)$$

and Σ is an Hermitian non-negative-definite matrix. ${\tilde{w}}_{i}$ is dependent of Γ with Assumptions 1 and 2. Therefore, we have:

(40) $$E\left[{∥{\tilde{w}}_{i-1}∥}_{\Gamma }^{2}\right]=E\left[{∥{\tilde{w}}_{i-1}∥}_{E[\Gamma ]}^{2}\right]$$

Let

(41) $$\gamma =vec\left\{E\left[\Gamma \right]\right\}$$

and

(42) $$\sigma =vec\left\{\Sigma \right\}$$

where vec(.) is the transpose of the vectorization of a matrix. The Equation (40) can be rewritten to follow equation with (41), (42):

(43) $$E\left[{∥{\tilde{w}}_{i}∥}_{\sigma }^{2}\right]=E\left[{∥{\tilde{w}}_{i-1}∥}_{\gamma }^{2}\right]+E\left[s_{i}^{T}KC^{T}\Sigma \mathrm{CK}s_{i}\right]+2{\left(\mathrm{XQ}w^{0}\right)}^{T}\Sigma C\left(I_{\mathrm{MN}}-\mathrm{KH}+\mathrm{XQ}\right)E\left[{\tilde{w}}_{i-1}\right]+\mathrm{CXQ}w^{0}$$

The vectorization operator denoted by Reference [42] is:

(44) $$vec\left\{ABC\right\}=\left(C^{T}⊗A\right)vec\left\{B\right\}$$

Taking expectation and vectorization operations with (38), (41), (42), we have

(45) γ=δσ

where

(46) $$\delta =E\left[\left(I_{\mathrm{MN}}-\mathrm{KH}+\mathrm{XQ}\right)⊗\left(I_{\mathrm{MN}}-\mathrm{KH}+\mathrm{XQ}\right)\right]Z$$

(47) $$Z=E\left[C^{T}⊗C^{T}\right]$$

Based on the relationship of the matrix trace and the vectorization operator [42], we have

(48) $$tr\left\{A^{T}B\right\}=vec^{T}\left\{B\right\}vec\left\{A\right\}$$

Σ is symmetric and deterministic, and we obtain

(49) $$E\left[s_{i}^{T}KC^{T}\Sigma \mathrm{CK}s_{i}\right]=vec^{T}\left\{V\right\}Z\sigma$$

where $V=KE\left[s_{i}s_{i}^{T}\right]K$. According to A.1 and A.2, V can be evaluated as

(50) $$V=blockdiag\left\{\eta^{2}s_{1}^{2}\left(w_{1}^{0}\right),\ldots ,\eta^{2}s_{K}^{2}\left(w_{K}^{0}\right)\right\}$$

Substitution of (45) and (50) into (43) has

(51) $$E\left[{∥{\tilde{w}}_{i}∥}_{\sigma }^{2}\right]=E\left[{∥{\tilde{w}}_{i-1}∥}_{\delta \sigma }^{2}\right]+vec\left\{V\right\}Z\sigma +2{\left(\mathrm{XQ}w^{0}\right)}^{T}\Sigma C\left(I_{\mathrm{MN}}-\mathrm{KH}+\mathrm{XQ}\right)E\left[{\tilde{w}}_{i-1}\right]+\mathrm{CXQ}w^{0}$$

the recursion of Equation (51) is stable and convergent if the matrix δ is stable. δ can be approximated as

(52) $$\delta \approx \left[\left(I_{\mathrm{MN}}-\mathrm{KH}+\mathrm{XQ}\right)⊗\left(I_{\mathrm{MN}}-\mathrm{KH}+\mathrm{XQ}\right)\right]Z$$

We know that all the entries of Z are non-negative and all its columns sum up to unity. From the above equation, the stability of δ is in accordance with the stability of $I_{\mathrm{MN}}-\mathrm{KH}+\mathrm{XQ}$. Therefore, choosing the step-size lined with the Equation (37) can keep the proposed algorithm stable in the mean-square sense.

## 4. Simulation

In this section, we validate the performance of the proposed algorithm over multitask sensor networks in two scenarios: a Gaussian environment and an impulsive noise environment. The noise is assumed to be generated by a Gaussian mixture distribution, which is commonly used in signal processing [43,44]:

(53) $$p_{n_{i}}=\left(1-v_{i}\right)N\left(0,\sigma_{1}^{2}\right)+v_{i}N\left(0,\sigma_{2}^{2}\right)$$

where $N\left(0,\sigma_{i}^{2}\right)\left(i=1,2\right)$ is the Gaussian distribution with zero-mean and variance $\sigma_{i}^{2}$. And $\sigma_{2}^{2}$ is set to much larger than $\sigma_{1}^{2}$, which can generate the impulsive noise.

More frequent impulses are achieved with an increase of $v_{i}$, especially

(54) $$\left\{\begin{array}{c} \;\mathrm{if}\;\nu_{i}=0\to Gaussian\\ \;\mathrm{if}\;\nu_{i}\neq 0\to Impulsive. \end{array}\right.$$

Increasing $\nu_{i}$ leads to more frequent impulses.

**Figure 2:**
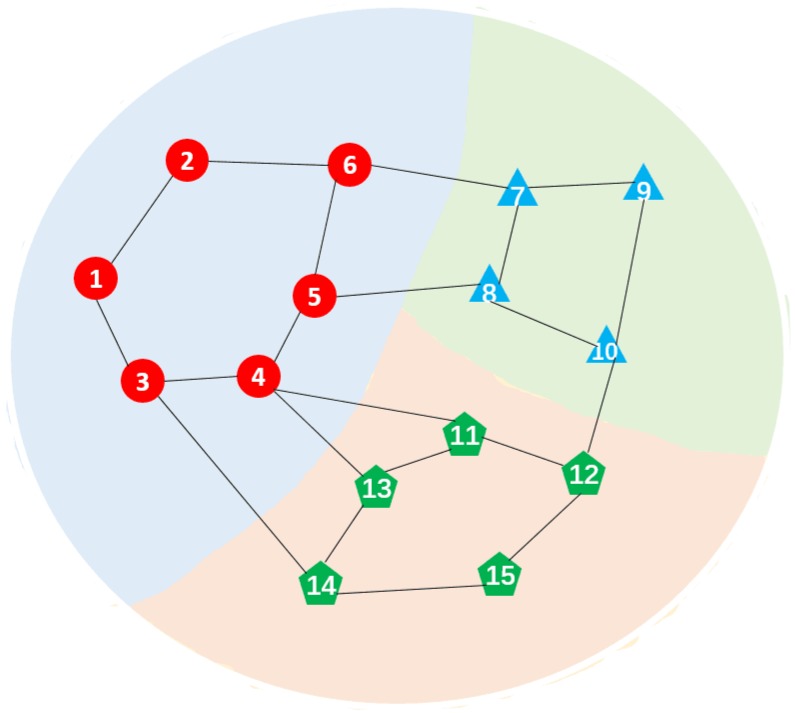
Network topology.

We consider a fully connected sensor network with 15 nodes. The network topology and cluster structures are demonstrated in Figure 2. From the network topology, we can easily find that nodes 1 to 6 belong to the first cluster. Meanwhile, nodes 7 to 10 compose the second cluster and nodes 11 to 15 are in the third cluster.

**Figure 3:**
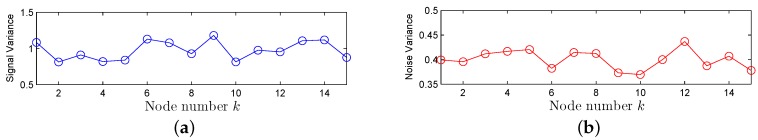
The variances of the input signal (**a**) and noise (**b**).

Input variances and noise variances based on Assumptions 1 and 2 are depicted in Figure 3.

**Figure 4:**
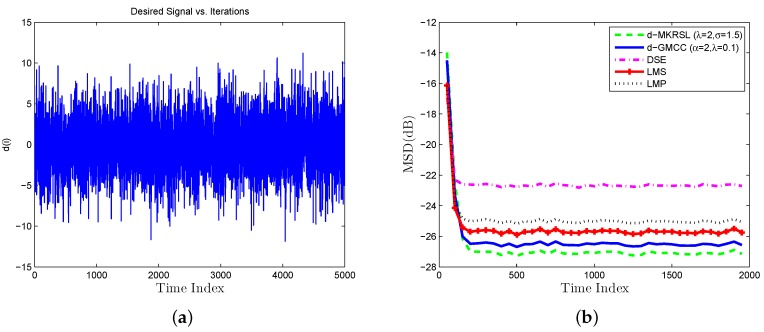
Gaussian noise environment (**a**) desired signal (**b**) transient network MSD(dB).

Scenario 1 (Gaussian noises Environment): As shown in Figure 3, the desired signal is a random process with a zero-mean Gaussian (i.i.d.) noise signal. In the experiment, system parameters are set with λ=2,σ=1.5 and the step-size is set with η=0.02. τ is a regularization parameter, which promotes similarities between the tasks of the neighboring cluster and is chosen τ=0.1. The learning curve of the mean square deviation(MSD) is defined as

(55) $$MSD=\frac{1}{K}\sum_{k=1}^{K}{∥w_{k}^{0}-w_{k,i}∥}_{2}^{2}$$

which is adopted for performance comparison. d(i) is the average value of $d_{k,i}$ for all nodes *k* at time *i* in Figure 4a. We compare some related algorithms over multitask network, such as diffusion least mean p-power (D-LMP) [21], diffusion generalized maximum correntropy criterion algorithm (D-GMCC) [16], diffusion sign-error LMS (DSE-LMS) [22], D-LMS [12] and the proposed d-MKRSL algorithm in Figure 4b. The step-sizes of all algorithms are chosen after many experiments to ensure the same convergence speed, and other parameters for each algorithm are experimentally selected to achieve a desirable performance. From the above figure, we can conclude that the D-MKRSL algorithm outperforms other related algorithms in the Gaussian noise environment.

**Figure 5:**
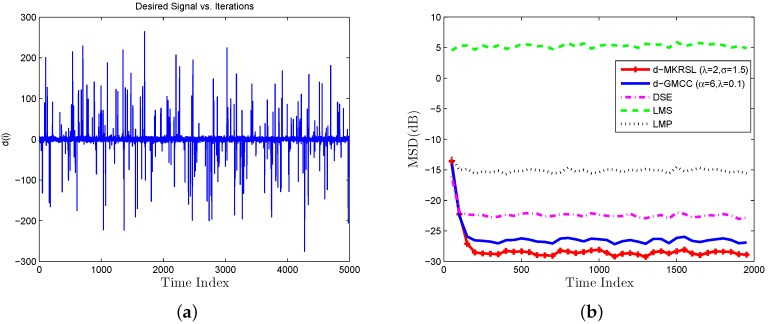
Impulsive interference environment of $v_{i}=0.05$ (**a**) desired signal (**b**) transient network MSD(dB).

**Figure 6:**
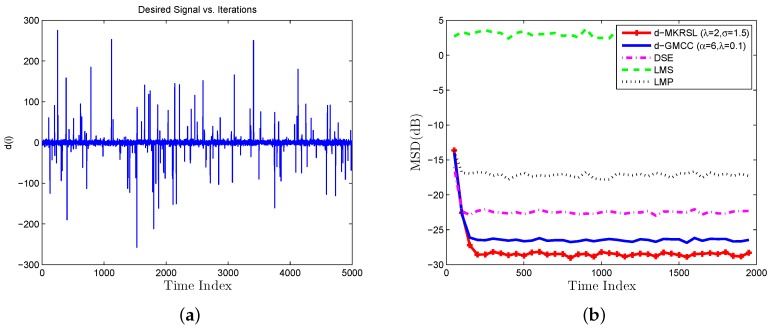
Impulsive interference environment of $v_{i}=0.03$ (**a**) desired signal (**b**) transient network MSD(dB).

Scenario 2 (Impulsive noise Environment): The impulsive noise model (54) is adopted to depict the distribution of impulsive interference in the experiment. We now test the influence of the impulsive interference on the performance of the algorithms mentioned above. In Figure 5a and Figure 6a, the desired signals are plotted with $v_{i}=0.05,0.03$ impulsive noise. The corresponding performance of the algorithms in the impulsive noise environment is plotted in Figure 5b and Figure 6b. The value of the parameters α and λ for D-GMCC are selected to achieve the best performance in both the Gaussian and impulsive noise environments. We can observe that the proposed D-MKRSL algorithm is robust and also shows superior performance compared with other related algorithms in the impulsive noise environment.

**Figure 7:**
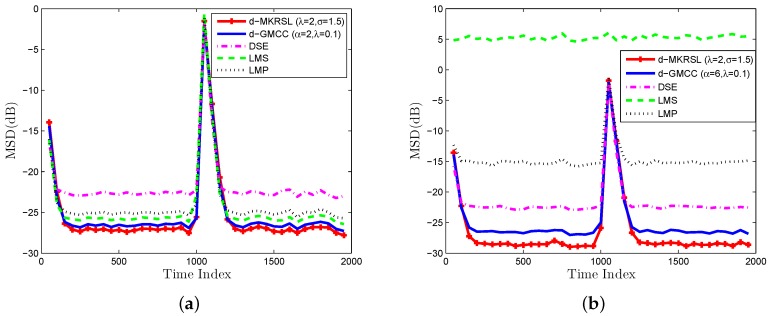
MSD learning curves in a non-stationary environment (**a**) Gaussian environment (**b**) Impulsive Interference.

Furthermore, we consider the performance of the algorithm in a nonstationary scenario and the unknown vector $w_{k}^{0}$ is assumed to change at time 1000. From the convergence curves in Figure 7, it can be easily observed that the proposed algorithm maintains a desirable performance even in the presence of sudden changes of an unknown vector.

**Figure 8:**
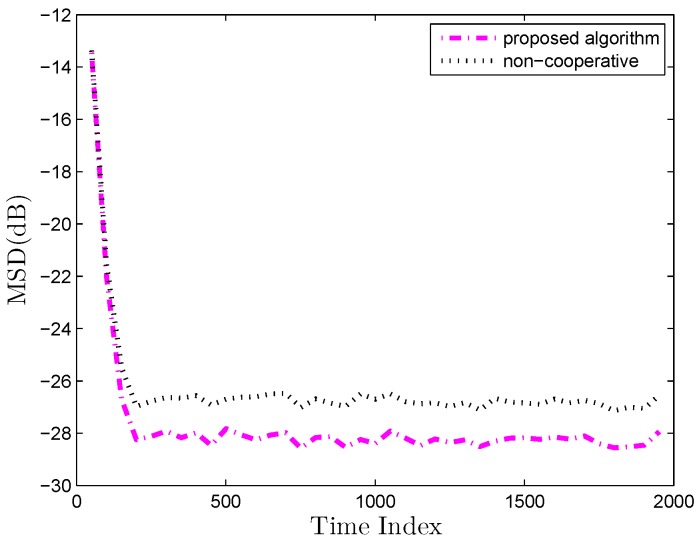
Network MSD comparison over multitask environment.

Another important aspect is how the correlation of tasks influence the estimation performance. First, we investigate whether the proposed algorithm can promote performance by utilizing the relatedness of tasks compared with non-cooperative strategy. Figure 8 compares the D-MKRSL algorithm with a non-cooperative strategy over a multitask network at identical relatedness of tasks. It is clear that utilizing the relatedness of tasks is beneficial to improve the performance of estimation. Next, the impact of the similarity of tasks on performance is studied. According to Reference [35], the optimum mean vector is assumed to uniformly distribute on a circle of radius *r* centered at $w_{k}^{0}$. The bigger the value of *r* is, the smaller the correlation of the tasks will be. Optimum parameter vectors over the multitask network will be different but related based on the model. The multitask estimation model can be expressed as:

(56) $$\begin{array}{r} w_{k}^{0}=w^{0}+r\left(\begin{array}{c} \cos\theta_{k}\\ \sin\theta_{k} \end{array}\right)\\ \theta_{k}=2\pi \left(k-1\right)/N+\pi /8 \end{array}$$

**Figure 9:**
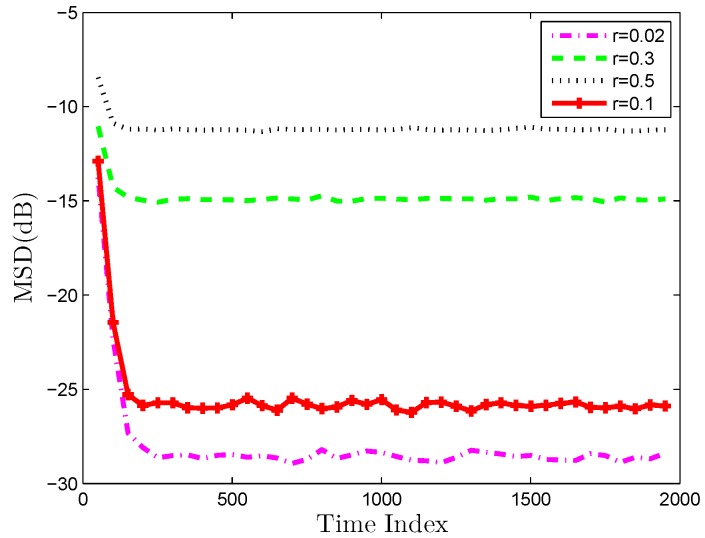
Network MSD comparison with different *r* value.

Figure 9 demonstrates that the performance of the algorithms will be improved with the increasing similarity.

## 5. Conclusions

In this work, we consider the problem of distributed estimation over multitask sensor networks. Then, the D-MKRSL algorithm is proposed and can achieve a desirable performance. Through theoretical analysis, a sufficient condition for ensuring the stability of the D-MKRSL algorithm is obtained. Compared with related algorithms, the simulation results show that the D-MKRSL algorithm has better performance in both Gaussian and impulsive noise environments. Furthermore, we uncover the relationship between the relatedness of tasks and estimation performance. It is demonstrated that the performance is improved with a higher correlation among tasks by cooperation strategy.
